# Prevalence of clinically-evident congenital anomalies in the Western highlands of Guatemala

**DOI:** 10.1186/s12978-020-01007-5

**Published:** 2020-11-30

**Authors:** Lester Figueroa, Ana Garces, K. Michael Hambidge, Elizabeth M. McClure, Janet Moore, Robert Goldenberg, Nancy F. Krebs

**Affiliations:** 1 Instituto de Nutrición de Centro América y Panamá (INCAP), Guatemala City, Guatemala; 2 Department of Pediatrics, University of Colorado, Denver, CO USA; 3 RTI International, Durham, NC USA; 4 Department of Obstetrics/Gynecology, Columbia University, New York, NY USA

**Keywords:** Congenital anomalies, ICD 10, Guatemala, Global network

## Abstract

**Background:**

Congenital anomalies are a significant cause of death and disability for infants, especially in low and middle-income countries (LMIC), where 95% of all deaths due to anomalies occur. Limited data on the prevalence and survival of infants with congenital anomalies are available from Central America. Estimates have indicated that 53 of every 10,000 live births in Guatemala are associated with a congenital anomaly. We aim to report on the incidence and survival of infants with congenital anomalies from a population-based registry and classify the anomalies according to the International Classification of Disease, Tenth Revision (ICD-10).

**Methods:**

We conducted a planned secondary analysis of data from the Maternal Newborn Health Registry (MNHR), a prospective, population-based study carried out by the Global Network for Women’s and Children’s Health Research in seven research sites. We included all deliveries between 2014 and 2018 in urban and rural settings in Chimaltenango, in the Western Highlands of Guatemala. These cases of clinically evident anomalies were reported by field staff and reviewed by medically trained staff, who classified them according to ICD – 10 categories. The incidence of congenital anomalies and associated stillbirth, neonatal mortality, and survival rates were determined for up to 42 days.

**Results:**

Out of 60,142 births, 384 infants were found to have a clinically evident congenital anomaly (63.8 per 10,000 births). The most common were anomalies of the nervous system (28.8 per 10,000), malformations and deformations of the musculoskeletal system (10.8 per 10,000), and cleft lip and palate (10.0 per 10,000). Infants born with nervous system anomalies had the highest stillbirth and neonatal mortality rates (14.6 and 9.0 per 10,000, respectively).

**Conclusions:**

This is the first population-based report on congenital anomalies in Guatemala. The rates we found of overall anomalies are higher than previously reported estimates. These data will be useful to increase the focus on congenital anomalies and hopefully increase the use of interventions of proven benefit.

**Trial registration:**

ClinalTrial.gov ID: NCT01073475.

## Background

Congenital anomalies are structural or functional anomalies that occur during intrauterine life and can be identified prenatally, at birth, or later in life [1, 2]. Congenital anomalies account for approximately 7% of neonatal deaths and 25.3–38.8 million disability-adjusted life-years (DALYs) worldwide [3]. The World Health Organization (WHO) and a recent global burden of disease (GBD) study report that congenital anomalies rank 17th among the causes of disease burden [4].

Congenital anomalies are a significant cause of stillbirths, neonatal mortality, and disability in low- and middle-income country (LMIC) settings, where 95% of all deaths occur. The median prevalence of nervous system defects in Latin America is reported as 11.5 per 10,000 births [5].

However, in Central America, and specifically in Guatemala, data related to congenital anomalies are limited. It is estimated that only one-third of congenital anomalies are reported due to the lack of access to prenatal care, properly trained staff, or even access to the proper technology to identify congenital anomalies. From available Ministry of Health (MOH) data, an estimated 2% of neonatal deaths are caused by congenital anomalies, with 67% of the reported anomalies identified as nervous system defects and 15% as cleft lip and palate [6]. Another report from a Guatemalan Western Highlands hospital-based registry found a nervous system defect rate of 27 per 10,000 live births, which is 2.5 times higher than the median reported for Latin America [7]. It is likely that the role of anomalies is under-estimated, since rural areas lack access to trained health personnel to identify and report congenital anomalies properly. This is of particular concern in this region and in LMICs in general, as many anomalies associated with environmental exposures, nutrient deficits and even some viral infections are potentially preventable [8, 9].

To determine the prevalence of stillbirth and neonatal mortality rates and survival for infants with clinically evident congenital anomalies in the Western Highlands of Guatemala, we conducted a secondary analysis from the Global Network Maternal Newborn Health Registry (MNHR) using the International Classification of Disease (ICD-10) to analyze data collected by trained auxiliary nurses in a standardized way.

## Methods

The MNHR is a prospective, population-based registry of pregnancies and deliveries conducted under the auspices of the Global Network for Women’s and Children’s Health Research (GN), a multi-country research network funded by the *Eunice Kennedy Shriver* National Institute of Child Health and Human Development (NICHD), of the United States. This registry has been described elsewhere [10].

The main objectives of the MNHR are to quantify and understand the trends in pregnancy services and outcomes over time in defined, low-resource geographic areas and provide statistics on stillbirths, neonatal and maternal mortality as the basis of understanding and improving health outcomes during pregnancy and childhood.

All pregnant women in participating clusters are registered, and their outcomes tracked through 6 weeks post-delivery. For this study, neonatal data (including clinically evident congenital anomalies reported by the delivery attendant) are collected at delivery and 42 days post-partum from the delivery attendant, other providers, and the mother or family. In case of a stillbirth or infant death, a cause of death form is completed to document the clinical cause [11]. For this analysis, we did not consider medical terminations of pregnancies because they are illegal in Guatemala, unless the mother’s life is at risk [12].

The GN’s Guatemalan site is situated in Chimaltenango, in the Western Highlands of Guatemala. In this area, 78% of the population identify themselves as Mayan descendants, and about 45% live in rural areas [13]. Chimaltenango has only one local referral hospital with limited diagnostic tools and do not perform level 3 ultrasounds or computed tomography (CT) scans.

From January 2014 to December 2018, the study staff collected pregnancy outcome data, including data on congenital anomalies. To record anomalies, the study staff received training on identifying and categorizing congenital anomalies. In the first 48 h after delivery and up to 42 days of life, research staff conducted a clinical examination of the baby, whenever possible, reviewed the medical charts for those births delivered at a hospital, and reviewed the available death certificates. Once all the information was collected in the field, the case report for each congenital anomaly reported was then reviewed by local experts. Using the available data, staff classified these congenital anomalies using the ICD-10 criteria into categories and subcategories [14]. We also calculated the stillbirth and neonatal mortality rates associated with congenital anomalies per 10,000 births.

## Ethics

This study was reviewed and approved by the institutional review boards of participating institutions, including the University of Colorado and the Universidad Francisco Marroquin in Guatemala. All women provided informed consent before enrollment in the study.

## Results

We identified 384 infants with a clinically-evident congenital anomaly among the 60,142 births enrolled from January 2014 to December 2018. Of those, 60% of the births had data collected from a local public hospital facility, 35% were home births, and about 3% occurred in a private facility. The annual rates of reported congenital anomalies varied between 45.1 per 10,000 births in 2016 and 100.1 per 10,000 births in 2014, with an overall rate of 63.8 per 10,000 births (Tables 1 and 2). The most common congenital anomalies reported were nervous system defects such as anencephaly (80 cases), hydrocephaly (60 cases), and spina bifida (33 cases), with an overall rate of 28.8 per 10,000 births (173 cases). Malformations and deformations of the musculoskeletal system, such as deformities of hands and feet and gastroschisis (rate 10.8 per 10,000 births, 65 cases) and cleft lip and cleft palate (rate of 10.0 per 10,000 births, 60 cases) were also identified. Other anomalies such as Down syndrome, gastrointestinal malformations, and cardiac malformations were observed less frequently, with rates ranging from 0.2 to 5.8 per 10,000 births.

**Table 1:** Rates of Congenital Anomalies Reported in Chimaltenango Per Year Per 10,000 Births

YEAR	TOTAL OF BIRTHS	TOTAL OF NUMBER OF CONGENITAL ANOMALIES	RATE OF CONGENITAL ANOMALIES PER 10,000 Births
2014	12,287	123	100.1
2015	12,861	71	55.2
2016	12,646	57	45.1
2017	11,847	69	58.2
2018	10,501	64	60.9
TOTAL	60,142	384	63.8

GN06 DATA MNHR PROTOCOL

**Table 2:** Type of Congenital Anomalies Reported From 2014 to 2018

ICD-10 Classification	Years					Totals		
	2014	2015	2016	2017	2018	N	% of CA	Prevalence per 10,000 births
Q00-Q07 Congenital malformations of the nervous system	34	40	28	42	29	173	45.1%	28.8
Q10-Q18 Congenital malformations of eye, ear, face, and neck	4	1	0	0	0	5	1.3%	0.8
Q20-Q28 Congenital malformations of the circulatory system	4	3	2	4	3	16	4.2%	2.7
Q30-Q34 Congenital malformations of the respiratory system	1	1	0	0	1	3	0.8%	0.5
Q35-Q37 Cleft lip and cleft palate	22	5	8	9	16	60	15.6%	10.0
Q38-Q45 Other congenital malformations of the digestive system	7	8	3	3	4	25	6.5%	4.2
Q50-Q56 Congenital malformations of genital organs	0	0	0	0	1	1	0.3%	0.2
Q60-Q64 Congenital malformations of the urinary system	0	1	0	0	0	1	0.3%	0.2
Q65-Q79 Congenital malformations and deformations of the musculoskeletal system	33	9	11	5	7	65	16.9%	10.8
Q90-Q99 Chromosomal abnormalities, not elsewhere classified	18	3	5	6	3	35	9.1%	5.8
Totals	123	71	57	69	64	384	100.0%	63.8

GN06 DATA MNHR PROTOCOL

Stillbirths and neonatal mortality rates due to congenital anomalies varied substantially by the type of anomaly. The overall rate of stillbirths attributed to congenital anomalies was 16.6 per 10,000 births. The rate of stillbirths associated with a nervous system defect was 14.6 per 10,000 births. Stillbirths with congenital malformations and deformations of the musculoskeletal system were identified in 0.7 per 10,000 births, and stillbirths with cleft lip and cleft palate were identified in 0.2 per 10,000 births.

The neonatal mortality rate reported at 42 days due to congenital anomalies for the overall population was 22 per 10,000 live births. Nine of every 10,000 live births were born with a nervous system defect and died before 42 days of life. Neonatal mortality attributable to malformations and deformations of the musculoskeletal system was 4.2 per 10,000 births, and cleft lip and cleft palate was associated with one neonatal death per 10,000 live births.

In comparing survival rates to 42 days among infants with congenital anomalies, the lowest survival was 18.5% among babies with a nervous system defect. (50.9% of the deaths were stillbirths and 30.6% were infants reported dead by 42 days). Of babies with congenital malformations and deformations of the musculoskeletal system, 55.4% survived to day 42 (6.2% were reported as stillbirths and 38.5% were reported dead at 42 days). Of those with a cleft lip and/or cleft palate, 88.3% survived (only 1.7% of the babies were reported as stillbirth and 10% were reported dead at 42 days) (Tables 2 and 3).

**Table 3:** Congenital Anomalies: Stillbirth, Neonatal Mortality and Survival Rates from 2014 to 2018

			Infants with congenital anomalies alive at 42 days		Infants with congenital anomalies stillborn			Infants with congenital anomalies reported dead at 42 days		
ICD-10 Classification	# Anomaly	Rate per 10,000 births	#	% Survival	Stillbirths	%	Rate per 10,000 births	Dead at 42 Days	%	Rate per 10,000 live births
Q00-Q07 Congenital malformations of the nervous system	173	28.8	32	18.5%	88	50.9%	14.6	53	30.6%	9.0
Q10-Q18 Congenital malformations of eye, ear, face, and neck	5	0.8	3	60.0%	2	40.0%	0.3	0	0.0%	0.0
Q20-Q28 Congenital malformations of the circulatory system	16	2.7	4	25.0%	1	6.3%	0.2	11	68.8%	1.9
Q30-Q34 Congenital malformations of the respiratory system	3	0.5	1	33.3%	0	0.0%	0.0	2	66.7%	0.3
Q35-Q37 Cleft lip and cleft palate	60	10.0	53	88.3%	1	1.7%	0.2	6	10.0%	1.0
Q38-Q45 Other congenital malformations of the digestive system	25	4.2	14	56.0%	0	0.0%	0.0	11	44.0%	1.9
Q50-Q56 Congenital malformations of genital organs	1	0.2	1	100.0%	0	0.0%	0.0	0	0.0%	0.0
Q60-Q64 Congenital malformations of the urinary system	1	0.2	0	0.0%	0	0.0%	0.0	1	100.0%	0.2
Q65-Q79 Congenital malformations and deformations of the musculoskeletal system	65	10.8	36	55.4%	4	6.2%	0.7	25	38.5%	4.2
Q90-Q99 Chromosomal abnormalities, not elsewhere classified	35	5.8	10	28.6%	4	11.4%	0.7	21	60.0%	3.6
Total	384	63.8	154	40.1%	100	26.0%	16.6	130	33.9%	22.0

GN06 DATA MNHR PROTOCOL

## Discussion

Data on the prevalence of congenital anomalies are needed to design effective strategies for their prevention and management. In this secondary analysis of a population-based registry, we found rates of clinically evident congenital anomalies reported by field staff to be higher than reported in the literature, including nervous system defects (28.8 per 10,000 births) compared to the regional and national estimates (3.3–27.9 with an average of 11.5 per 10,000 births in the American region) [15].

Many nervous system defects are preventable with folic acid supplementation before pregnancy. Although Guatemala’s national data do not indicate significant micronutrient deficencies, data from populations similar to the one we studied indicate this possibility [16]. Rates of cleft lip and cleft palate of 10 per 10,000 births were also found to be higher than previous reports from other Guatemalan regions, with reported rates between 4.7 and 18.9 per 10,000 births [17].

Among those babies born alive with congenital anomalies, the survival rate at 42 days of life was 40.1%. Babies born with cleft lip and cleft palate had the highest survival rate at 88.3% compared to 18.5% of those with defects of the nervous system. Early access to surgery for this defect improves survival for these babies.

This study had a number of limitations. First, we were only capable of capturing the clinically-evident congenital anomalies. In addition, the population of this sub-study had little or no access to diagnostic technology to determine defects prenatally, to refine the diagnoses or to capture those that were not physically evident at delivery. Additionally, sophisticated techniques such as genetic screening were also not routinely available. Thus, congenital heart disease and other anomalies that require various technologies for diagnosis were likely to be underestimated in this study.

However, this study did have a number of strengths that improve upon existing data on this topic. This was the first attempt to systematically report congenital anomalies using ICD-10 with data gathered at the community level and available within health services and collected by MNHR staff. Thus, we were able to capture higher rates of anomalies than those currently reported in the literature for this area [2, 3, 18].

## Conclusions

This is the first population-based study to report clinically evident congenital anomalies diagnosed by health personnel in Guatemala. Nervous system defects were the most common anomaly reported with the highest neonatal mortality and stillbirth rates, while cleft lip and cleft palate were associated with lower mortality rates.

These data will be useful to better understand congenital anomalies and the importance of the prenatal care and other interventions that are likely to be beneficial in reducing congenital anomalies. Interventions including family planning, a healthy diet for women of reproductive age, better control of infections in pregnancy as well as decreasing exposure to ambient contaminants like agricultural pesticides may all help reduce risk of congenital anomalies. Finally, with better diagnostic tools, all babies born with a congenital anomalies should have early access to medical care and appropriate surgery to reduce mortality.

## Data Availability

The datasets used and/ or analyzed during this study are available from the corresponding author on reasonable request.

## Abbreviations

DALYs: Disability-adjusted life-years
GBD: Global burden of disease
GN: Global network for women’s and children’s health research
ICD-10: International classification of disease, tenth revision
LMIC: Low and middle-income countries
MNHR: Maternal newborn health registry
MOH: Ministry of health
NICHD: Eunice Kennedy Shriver national institute of child health and human development

## References

[1] WHO. Birth defects Report by the Secretariat [Internet]. 2010 [cited 2020 Sep 13]. Available from: https://apps.who.int/gb/ebwha/pdf_files/WHA63/A63_10-en.pdf?ua=1.

[2] WHO. Congenital anomalies news room [Internet]. 7 September 2016 [cited 2020 Sep 13]. Available from: https://www.who.int/news-room/fact-sheets/detail/congenital-anomalies.

[3] Sitkin NA, Ozgediz D, Donkor P, Farmer DL. Congenital anomalies in low- and middle-income countries: the unborn child of global surgery. World J Surg. 2015;39(1):36–40. 10.1007/s00268-014-2714-9. 10.1007/s00268-014-2714-9 PMC4300430 25135175

[4] Stevens G, Mahanani R, Ho J, Fat DM, Hogan D. WHO methods and data sources for global burden of disease estimates 2000–2016 [internet]. 2000. Available from: http://www.who.int/gho/mortality_burden_disease/en/index.html.

[5] Zaganjor I, Sekkarie A, Tsang BL, Williams J, Razzaghi H, Mulinare J, Sniezek JE, Cannon MJ, Rosenthal J. Describing the Prevalence of Neural Tube Defects Worldwide: A Systematic Literature Review. PLoS One. 2016;11(4):e0151586. 10.1371/journal.pone.0151586. 10.1371/journal.pone.0151586 PMC4827875 27064786

[6] Ministerio de Salud Publica y Asistencia Social de Guatemala. Memoria de Labores 2018 [Internet]. Centro Nacional de Epidemiologia. 2018 [cited 2020 Sep 13]. p. 609. Available from: http://epidemiologia.mspas.gob.gt/informacion/estadisticas-vitales/memorias-1999-2018.

[7] Biswas Roshni, Tao Vivian. Congenital Anomalies in Guatemala: Causes and Actions [Internet]. 2016. https://publichealth.gwu.edu/content/congenital-anomalies-guatemala-causes-and-actions. [cited 2020 Sep 13]. Available from: https://publichealth.gwu.edu/content/congenital-anomalies-guatemala-causes-and-actions.

[8] Toufaily MH, Westgate MN, Lin AE, Holmes LB (2018). Causes of congenital malformations. Birth Defects Res.

[9] Semih Demirtaş M. The Pathogenesis of Congenital Anomalies: Roles of Teratogens and Infections. Congenit Anomalies Neonates - Clin Perspect [Working Title] [Internet]. IntechOpen; 2020 [cited 2020 Sep 13]. Available from: https://www.intechopen.com/online-first/the-pathogenesis-of-congenital-anomalies-roles-of-teratogens-and-infections.

[10] Goudar SS, Carlo WA, McClure EM, Pasha O, Patel A, Esamai F (2012). The maternal and newborn health registry study of the global network for women’s and children’s health research. Int J Gynecol Obstet.

[11] Garces AL, McClure EM, Pérez W, Hambidge KM, Krebs NF, Figueroa L (2017). The global network neonatal cause of death algorithm for low-resource settings. Acta Paediatr Int J Paediatr.

[12] Gobierno de Guatemala. CODIGO PENAL DE GUATEMALA [Internet]. Constitucion Politica de la republica. [cited 2020 Sep 13]. Available from: https://www.congreso.gob.gt/assets/uploads/info_legislativo/iniciativas/1542041414_5494.pdf.

[13] Gobierno de Guatemala. Censo Poblacional 2018 [Internet]. 2018 [cited 2020 Sep 13]. Available from: https://www.censopoblacion.gt/dondeestamos.

[14] WHO. ICD-10 Version:2016 [Internet]. 2016. 2016 [cited 2019 May 14]. Available from: https://icd.who.int/browse10/2016/en.

[15] Rosenthal J, Casas J, Taren D, Alverson CJ, Flores A, Frias J. Neural tube defects in Latin America and the impact of fortification: a literature review. Public Health Nutr. 2014;17(3):537–50. 10.1017/S1368980013000256. 10.1017/S1368980013000256 PMC4479156 23464652

[16] Mazariegos M, Martínez C, Mazariegos DI, Méndez H, Román AV, Palmieri M, Tomás V. Análisis de la situación y tendencias de los micronutrientes clave en Guatemala, con un llamado a la acción desde las políticas públicas. Washington, D.C: FHI 360/FANTA; 2016.

[17] Matute J, Lydick EA, Torres OR, Owen KK, Jacobsen KH. Prevalence of cleft lip and cleft palate in rural North-Central Guatemala. Cleft Palate Craniofac J 2015. 52:377–380. American Cleft Palate Craniofacial Association; [cited 2020 Sep 13]; Available from: 10.1597/13-347. 10.1597/13-347 25058118

[18] Chúa Carlos. ANOMALÌAS DEL TUBO NEURAL EN GUATEMALA [Internet]. Guatemala; 2004 [cited 2020 Sep 13]. Available from: http://biblioteca.usac.edu.gt/tesis/07/07_1440.pdf.

